# Characterization of Modified Magnetite Nanoparticles for Albumin Immobilization

**DOI:** 10.1155/2014/705068

**Published:** 2014-05-22

**Authors:** A. K. Bordbar, A. A. Rastegari, R. Amiri, E. Ranjbakhsh, M. Abbasi, A. R. Khosropour

**Affiliations:** ^1^Department of Chemistry, University of Isfahan, Isfahan 81746-73441, Iran; ^2^Department of Biotechnology, Faculty of New Science and Technology, University of Isfahan, Isfahan 81746-73441, Iran; ^3^Department of Molecular and Cell Biochemistry, Falavarjan Branch, Islamic Azad University, Isfahan, Iran

## Abstract

Magnetite Fe_3_O_4_ nanoparticles (NPs) were prepared by chemical coprecipitation method. Silica-coated magnetite NPs were prepared by sol-gel reaction, subsequently coated with 3-aminopropyltriethoxysilane (APTES) via silanization reaction, and then were activated with 2,4,6-trichloro-1,3,5-triazine (TCT) and covalently immobilized with bovine serum albumin (BSA). The size and structure of the particles were characterized by transmission electron microscopy (TEM), X-ray powder diffraction (XRD), and dynamic light scattering (DLS) techniques. The immobilization was confirmed by Fourier transform infrared spectroscopy (FT-IR). XRD analysis showed that the binding process has not done any phase change to Fe_3_O_4_. The immobilization time for this process was 4 h and the amount of immobilized BSA for the initial value of 1.05 mg BSA was about 120 mg/gr nanoparticles. Also, the influences of three different buffer solutions and ionic strength on covalent immobilization were evaluated.

## 1. Introduction

Magnetite nanoparticles are widely studied for their applications in various areas such as removal of organic and inorganic pollutants [1, 2], being contrast agent in magnetic resonance imaging (MRI) [3, 4], RNA and DNA purification [5], purification of antibodies [6], drug delivery [7, 8], and enzyme and protein immobilization [9–11]. Magnetite nanoparticles are produced by different methods. Several common methods include coprecipitation, microemulsion, thermal decomposition, and laser pyrolysis synthesis [12–16], in which, among these methods, coprecipitation is a facile and convenient way to synthesize magnetite nanoparticles.

Protein immobilization has wide application in many areas, such as immunological agglutination tests, biosensors, and bioseparation [17–19]. Isolation, separation, and purification of various types of the proteins and peptides are used in almost all branches of biosciences and biotechnologies. The basic principle of batch magnetic separation is very simple [20]. The immobilization of bovine serum albumin (BSA) or other proteins on magnetite nanoparticles has unique advantages: (1) increased surface area to volume and subsequent increase in the amount of immobilized protein onto carrier, (2) easy separation of the reaction mixture under a magnetic field, and (3) biocompatibility and nontoxicity with superparamagnetic properties. The magnetite nanoparticles (NPs) need to be stabilized in the carrier liquid because they tend to agglomerate. In order to prevent this problem, the Fe_3_O_4_ NPs are coated with a surfactant or a polymer [4, 21–24]. At pH range between 4 and 8, the surface charge density is low then; it is difficult to break up the agglomerations formed. For serum albumin at the high and low pH range, *ζ*-potential shows the highest values; therefore, sonication should be strong enough to break the weak bonding and prevent agglomeration [25].

According to the type of linkage of the protein, the carrier-binding immobilization is classified into three categories (adsorption, ionic, and covalent immobilization). The covalent immobilization can lead to irreversible binding of protein on different carriers. Bovine serum albumin (BSA) was covalently immobilized onto magnetite nanoparticles by different agents [26–29]. Human serum albumin was covalently immobilized on modified magnetic NPs so that it is significant for its magnetic applications in various bioprocesses, biomedical devices, and biomedicine [30].

Previously, we studied immobilization of porcine pancreas lipase (PPL) on chemically modified magnetite nanoparticles [31]. In this study, the silica-coated magnetite nanoparticles were synthesized. The silica-coated Fe_3_O_4_ nanoparticles is treated with 3-aminopropyltriethoxysilane (APTES) to yield the amino-functionalized magnetic nanoparticles (AFMNs); then with 2,4,6-trichloro-1,3,5-triazine as the coupling agent, BSA could be covalently attached to the surface of AFMNs. The characteristics of nanoparticles were investigated using various techniques. The size and morphology of the particles were characterized by dynamic light scattering (DLS) and transmission electron microscopy (TEM) techniques. Different stages of the synthesis of nanoparticles and immobilization were confirmed by Fourier transform infrared spectroscopy (FT-IR); and X-ray powder diffraction (XRD) technique revealed phase of the nanoparticles before and after immobilization. Optimization of immobilization conditions was done; and, also, the effects of different buffers and ionic strength on immobilization were efficiency investigated.

## 2. Material and Methods

### 2.1. Materials

Bovine serum albumin (BSA) and Coomassie Brilliant Blue G-250 were purchased from Sigma-Aldrich Co. Ferric chloride hexahydrate (FeCl_3_ · 6H_2_O), ferrous chloride tetrahydrate (FeCl_2_ · 4H_2_O), 3-(triethoxysilyl)-propylamin (APTES), ethanol (96%), tetraethoxysilane (TEOS), trichlorotriazine (TCT), and thetrahydrofuran (THF) were prepared from Merck. NaH_2_PO_4_ · 2H_2_O, Na_2_HPO_4_ · 12H_2_O, and tris-HCl were purchased from Merck and used for preparation of phosphate and tris-HCl buffers at pH = 7.5.

### 2.2. Synthesis of Magnetic Nanoparticles

The magnetite nanoparticles were prepared by chemical coprecipitation of Fe^3+^ and Fe^2+^ ions with a molar ratio of 2 : 1 [32]. The iron salts were dissolved in deionized water under nitrogen gas at 60°C, and then ammonia solution was added with vigorous stirring for 30–40 min. The pH of the solution kept constant in the range of 8-9 at this stage. After the addition of ammonia solution, the color of the solution was black. The magnetite precipitates were separated and washed several times with deionized water and once with ethanol.

### 2.3. Preparation of Silica-Coated Magnetite Nanoparticles

**Scheme 1:**
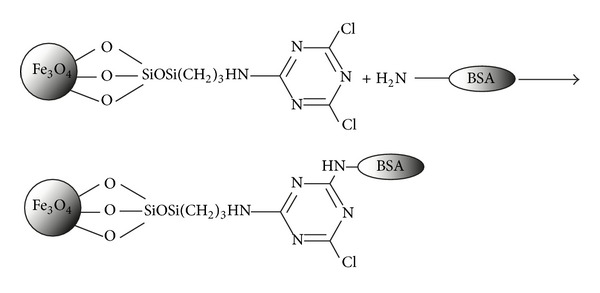
Covalent attachment of BSA on modified magnetite nanoparticles.

Silica-coated Fe_3_O_4_ nanoparticles were prepared by the hydrolysis of TEOS using sol-gel process. For this purpose, 0.145 gr Fe_3_O_4_ was mixed with 40 mL ethanol. The produced suspension was dispersed under ultrasonification for 10–15 min in the presence of a constant nitrogen flux. 6 mL water and 3 mL ammonium hydroxide solution were added to this suspension at room temperature, followed by the addition of 0.4 mL TEOS with stirring and the pH kept constant in the range of 9-10. The mixture was stirred for 5 h and subsequently, was washed with ethanol and water for several times and dried under vacuum at room temperature. The covalent attachment of BSA is shown in Scheme 1.

### 2.4. Nanoparticles Surface Modification

For the surface modification of the MNPs with 2,4,6-trichloro-1,3,5-triazine, an adaptation of the Wang and Liu et al. method was used [27]. First the silica surface was functionalized by 3-aminopropyltriethoxy silane. The obtained MNPs (240 mg) were treated with APTES (9 mL) in ethanol (6 mL) to introduce amino groups to the NPs surface. The mixture reacted at room temperature for 2 h, which was followed by heating at 50°C for 1.5 h. These NPs were washed successively with ethanol and THF. The obtained NPs were further reacted with TCT (750 mg) in THF (15 mL) at room temperature for 3 h under sonication for prevention of any agglomeration. The obtained NPs were washed with THF, ethanol, and Milli-Q water. Finally, the triazine functionalized MNPs were dried under vacuum at room temperature.

### 2.5. Albumin Immobilization

4.0 mg of the triazine functionalized MNPs was dispersed in 500 *μ*L of phosphate buffer (50 mM, pH = 7.5) and 500 *μ*L of BSA solution (1 mg/mL) was added to MNPs. The mixture was shaken at room temperature for 1–8 hours. The amount of BSA immobilized on MNPs was determined using the Bradford method.

### 2.6. Characterization

Size and morphology of magnetic nanoparticles were determined by transmission electron microscopy (Philips CM 10 HT-100 KV) and dynamic light scattering (PMX 200CS particle Metrix, Germany). X-ray diffraction measurement was recorded with X-ray diffractometer Bruker, D8ADVANCE ((Germany) using Cu K*α* radiation (*λ* = 0.1540 nm)). The FT-IR spectra were recorded by a Fourier transform infrared spectrophotometer (JASCO FT/IR-6300, Japan).

## 3. Results

### 3.1. Characterization of Nanoparticles

**Figure 1:**
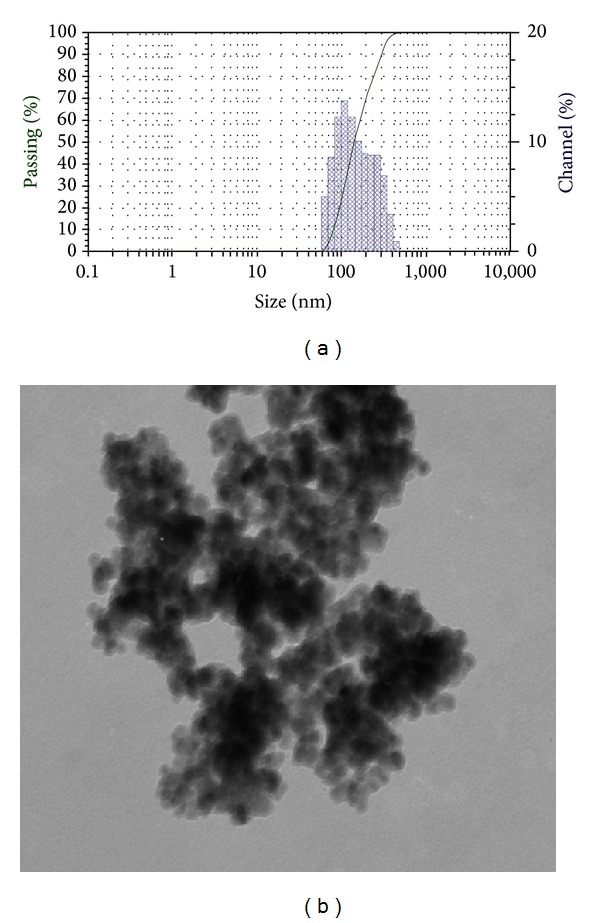
The DLS (a) and TEM (b) of silica-coated MNPs.

The size and morphology of the nanoparticles were studied by DLS and TEM techniques. The particle distribution bar chart of MNPs shows their quantities as well as the size distribution patterns as in Figure 1(a). The TEM picture of silica-coated magnetite nanoparticles were shown in Figure 1(b). The average particles size was estimated to be 17.5–21 nm.

**Figure 2:**
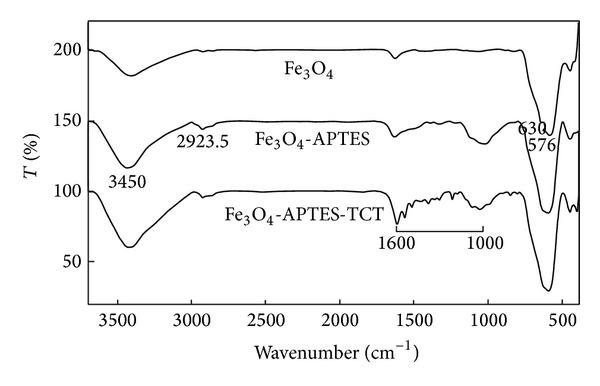
FT-IR spectra of the naked Fe_3_O_4_, APTES-modified, and TCT-APTES-modified magnetite nanoparticles.

The analysis of FT-IR spectroscopy confirmed different stages of nanoparticles and the binding of BSA on the surface of magnetite nanoparticles. The modification of magnetite nanoparticles was confirmed by using silica-uncoated nanoparticles because the peaks of TCT overlap with silica.  Figure 2 shows the FT-IR spectra of the naked Fe_3_O_4_, APTES-modified and TCT-APTES-modified magnetite nanoparticles.

**Figure 3:**
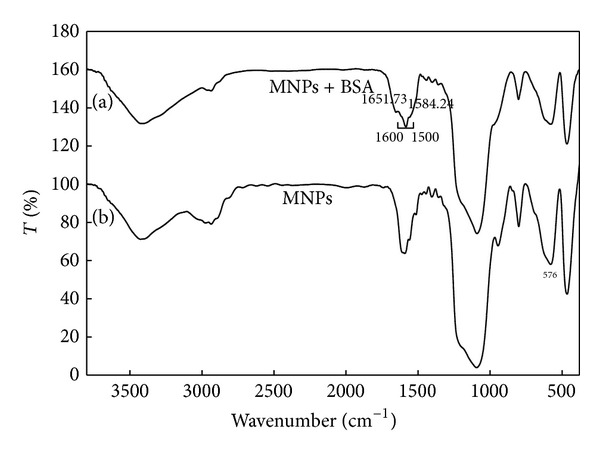
FT-IR spectra of the modified magnetite nanoparticles with (top) and without (bottom) bound BSA.

Figure 3 shows the FT-IR spectra of the modified particles with TCT and BSA-bound nanoparticles. The presence of magnetite NPs can be seen by wide strong absorption band between 580 and 630 cm^−1^ [33, 34], especially for Fe–O bond of bulk magnetite at 576 cm^−1^. Also, an absorption band was observed between 400 and 500 cm^−1^. C–H stretching vibration can be seen at around 2923.5 cm^−1^. The broad band at 3450 cm^−1^ can be referred to the N–H stretching vibration. The bands around 3430.7 and 1630 cm^−1^ were assigned to amid group. The fine peaks between 1000 and 1600 cm^−1^were assigned to TCT connection to NPs. The bands at 1017.3 and 1030 cm^−1^were assigned to the Si–O stretching vibration of APTES to the surface of magnetic NPs. Broad peak at 1500 to 1600 cm^−1^were assigned to C–N stretching vibration of BSA-MNPs.

**Figure 4:**
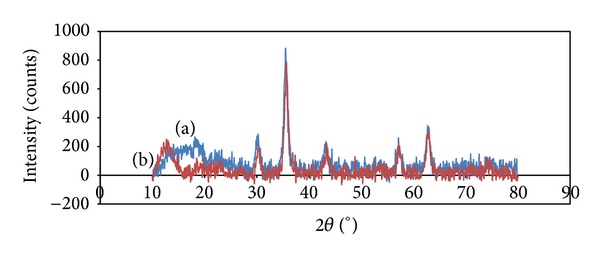
XRD patterns for magnetic nanoparticles with (a) and without (b) BSA.

The XRD patterns for the modified magnetite NPs without and with bound BSA were recorded in Figure 4. Six characteristic peaks for Fe_3_O_4_ (2*θ*) at 30.06°, 35.42°, 43.05°, 53.4°, 57.15°, and 62.79° were observed for both samples representing the nonoccurrence of any phase change during the immobilization of BSA.

### 3.2. BSA Immobilization Parameters

#### 3.2.1. Time of Immobilization

**Figure 5:**
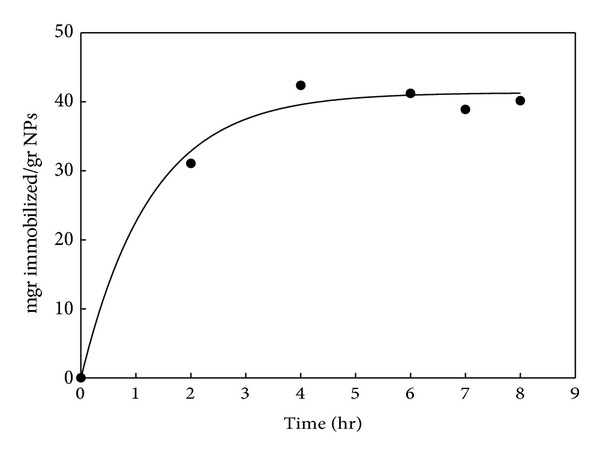
Effect of reaction time on the amount of immobilized BSA.

The amount of immobilized BSA as a function of reaction time (hour) is shown in Figure 5 and the corresponding data is listed in Table 1. It was found that, with increasing the reaction time from 0 to 4 h the amount of immobilized BSA increased and then remained constant.

#### 3.2.2. Concentration of Immobilization

**Figure 6:**
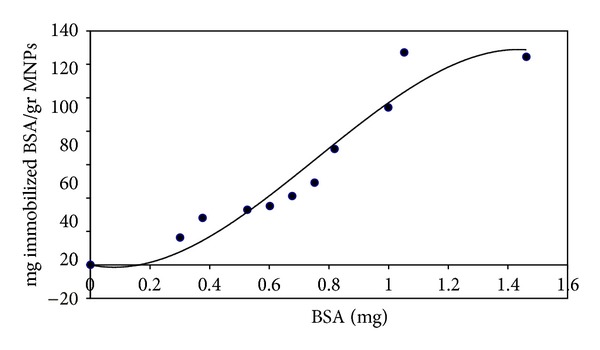
Effect of the initial amount of protein on the amount of immobilized BSA.

In this part, different amounts of the BSA (0.3 to 1.462 mg) were used for immobilization on 4 mg of Fe_3_O_4_ nanoparticles. The amount of immobilized BSA increases with increasing the initial value of BSA from 0.3 to 1.0 mg and remains constant after this initial value (see Figure 6). The immobilization time was 4 h for all measurements.

### 3.3. Effect of Various Buffer

Different buffers (PBS, tris-HCl, and phosphate buffer) were used to immobilize BSA on magnetite NPs.  Table 2 shows the immobilization percentage of protein for these different buffers. The percent of immobilized BSA is not identical for these various buffers with the same pH.

### 3.4. Effect of Ionic Strength

**Figure 7:**
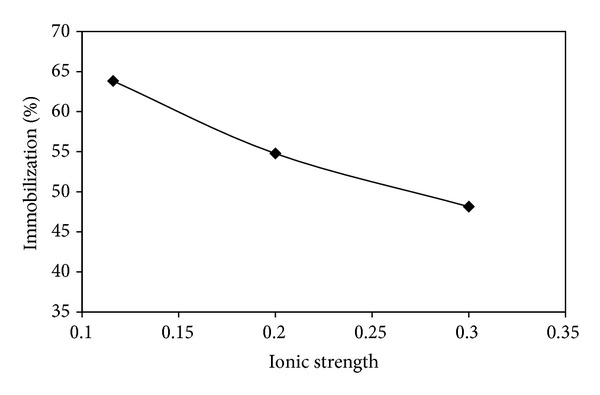
Effect of the ionic strength on the immobilization percentage.

Figure 7 shows the effect of ionic strength on the immobilized percentage of BSA. Phosphate buffer with different ionic strength (0.1–0.3) was obtained through solving different amounts of sodium chloride salt. Increasing of the ionic strength changes the immobilization percentage of BSA.

## 4. Discussion

In the first section of this study, BSA immobilization on magnetite nanoparticles was characterized by TEM, FT-IR, XRD, and DLS techniques. Figure 1(a) shows the particle distribution bar chart of MNPs as well as the size distribution patterns. 132.9 nm was estimated as the average particle size. The TEM picture of silica-coated magnetite nanoparticles was shown in Figure 1(b). This suggests the achievement of high specific areas of nanoparticles and their spherical shape and granular form.

In Figure 2, a peak at 576 cm^−1^ is a characteristic peak for Fe–O stretching band. The sharp peaks in the range of 1000–1600 cm^−1^ confirmed binding of TCT on the AFMNPs. After immobilization of BSA on the magnetic nanoparticles, the peaks at 1651.73 cm^−1^ and 1584.24 cm^−1^ (see Figure 3) corresponded to the amide I band (C = O stretching vibration) and amide II band (N–H bending vibration and the C–N stretching vibration) which reveals that the BSA is immobilized on modified nanoparticles. Therefore, the covalent immobilization of BSA can be concluded from these data.

The XRD pattern in Figure 4 shows that the binding process did not result in the phase change of Fe_3_O_4_. Also, using Debye-Scherrer equation, the particle size was calculated to be 9-10 nm.

In the second part of this study, some immobilization parameters such as time, concentration of BSA, effect of various buffer, and ionic strength were investigated. The amount of immobilized BSA reached the maximum after 4 h, representing the saturation of nanoparticles with BSA at this time (Figure 5). The weight ratio of immobilized BSA to MNPs at this time was 40 mg/g. According to Figure 6, the amount of immobilized BSA increases with increasing the initial value of BSA and remains constant at about 120 mg/gr NPs which is in agreement with previous study corresponding to BSA immobilization on coated MNPs with polymer [35]. The extent of immobilization reaches maximum at initial BSA value of 1.05 mg (Figure 6) that is related to saturation of MNPs at this value. This can be easily explained by considering the limited number of active sites on MNPs for attachment of BSA.

The extent of immobilization in phosphate buffer is higher than other examined buffers. This may be related to the deactivation of some active sites on MNPs. The effect of ionic strength on the extent of immobilization is shown in Figure 7. The results represent the decreasing of immobilization due to the increase of ionic strength that can be related to the reduction of electrostatic interactions between BSA and nanoparticles. Similar result has been observed previously [35].

## 5. Conclusion

Silica-coated magnetite nanoparticles were prepared by sol-gel reaction. The silanization of these coated particles by APTES and their activation by TCT were done, subsequently. BSA was covalently immobilized on these chemically modified MNPs. The size and structure of the particles were investigated by using TEM, XRD, and DLS methods. XRD results showed that the binding process did not induce any phase change on Fe_3_O_4_. Immobilization time for this process was 4 h and the amount of immobilized BSA was at about 120 mg per gram of nanoparticles. The PBS buffer showed the maximum extent of immobilization among the examined buffers. The obtained results also represent the decreasing of immobilization by increasing the ionic strength. The whole results in this article may be used for biological, biomedical, and biotechnological applications.

## Figures and Tables

**Table 1:** Weight ratio of immobilized BSA to MNPs (mg/g) versus immobilization time BSA on MNPs.

Weight ratio of immobilized BSA to MNPs (mg/g)	30	42	40	38	39

Immobilization time (h)	2	4	6	7	8

**Table 2:** Immobilized BSA value on MNPs in different buffers with pH = 7.5.

Various buffers	% immobilized BSA
Tris-HCl	43.92459
Phosphate buffer saline (PBS)	43.18039
Phosphate buffer	49.67127
